# Oral Plasmacytoma in a Dog

**DOI:** 10.3390/vetsci4040068

**Published:** 2017-12-14

**Authors:** Indira Pargass, Alissa Bally, Rod Suepaul

**Affiliations:** The School of Veterinary Medicine, University of the West Indies, Trinidad and Tobago; Alissa.Bally@sta.uwi.edu (A.B.); Rod.Suepaul@sta.uwi.edu (R.S.)

**Keywords:** neoplasia, oral, mandibular mass, plasmacytoma

## Abstract

A 6-year-old male Pit bull mix dog presented for bleeding from the mouth persisting for five days. A clinical evaluation revealed a 2 × 3 cm soft tissue mandibular mass at the crown of the first premolar, as well as a non-regenerative anemia and hyperproteinemia. Cytologic and histopathologic evaluations of the mass were compatible with an oral plasmacytoma.

## 1. Introduction

Plasma cell tumours are derived from cells of the B lymphocyte plasma cell lineage. Solitary collections of these neoplastic cells which originate in soft tissues or organs are referred to as extramedullary plasmacytomas (EMPs) [1,2]. EMPs comprise 2.4% of all neoplasms in dogs and are rare in cats [3]. In dogs, they are most commonly found in middle-aged to older dogs (mean 8–10 years), with American Cocker Spaniels, English Cocker Spaniels, Airedale Terriers, Kerry Blue Terriers, and Scottish Terriers being most commonly affected [4,5].

EMPs can develop from cutaneous or mucocutaneous tissues [6]. The most common locations of plasmacytomas in dogs are the oral cavity, ear pinnae, lips, chin, trachea, larynx, stomach, colon, and digits [3,4]. In a large case study of 751 EMPs, the most common location was the skin (86%), followed by the mucous membranes of the oral cavity (9%), and then the rectum and colon (4%). Other sites, such as the stomach, spleen, genitalia, eyes, uterus, and liver accounted for the remaining 1% of the cases [3].

This report describes a case of an oral plasmacytoma in a Pit bull mix dog in Trinidad and Tobago.

## 2. Materials and Methods

A 6-year-old male Pit bull mix dog was presented to the Veterinary Teaching Hospital at the School of Veterinary Medicine, University of the West Indies, Trinidad and Tobago, for a bleeding mass in the mouth which the owner noticed 5 days prior to presentation. 

Temperature, pulse, and respiratory rates were within normal limits, and there was no enlargement of the submandibular lymph nodes. In the oral cavity, there was a pink, round mass measuring 2 × 3 cm at the crown of the first premolar on the right lower jaw, and dental plaque was noted on all teeth. Radiographs of the thorax and skull were taken, and a fine needle aspirate of the mass was performed for cytological examination. Blood was also taken for a complete blood count (CBC). The cytology smears and blood smears were stained with Wright-Giemsa stain and examined. Eight days later, the owners decided to euthanize the dog, electing not to seek further treatment. At this time, samples of the oral mass were collected, placed in 10% buffered formalin, and sent for histopathological examination.

## 3. Results

### 3.1. Radiologic Findings

The radiological findings demonstrated osteolytic changes in the mandibular bone in the region underlying the mass. There was a well-circumscribed, irregular soft tissue opacity measuring approximately 2 × 3 cm at the rostral aspect of the right mandible. Premolar 1 on the right side of the jaw was also displaced, but it was not associated directly with the bone lysis. Thoracic views showed that there was no evidence of metastases to the lungs and no pathologic lung pattern. Osteophyte formation was found on the cranioventral border of the 4th thoracic vertebra (T4).

### 3.2. CBC Results

The complete blood count revealed a normocytic, normochromic, non-regenerative anemia (PCV 0.25 L/L, reference interval(RI) 0.37–0.55 L/L; MCV 65 fL, RI 60–77; MCHC 334; RI 320–360 g/L; reticulocyte count <1%) and hyperproteinemia (83 g/L, reference interval 55–76 g/L) due to a hyperglobulinemia (46 g/L; RI 18–36). Serum immunoelectrophoresis was not done to determine if the hyperproteinemia was due to a monoclonal gammopathy.

### 3.3. Cytologic Findings

**Figure 1:**
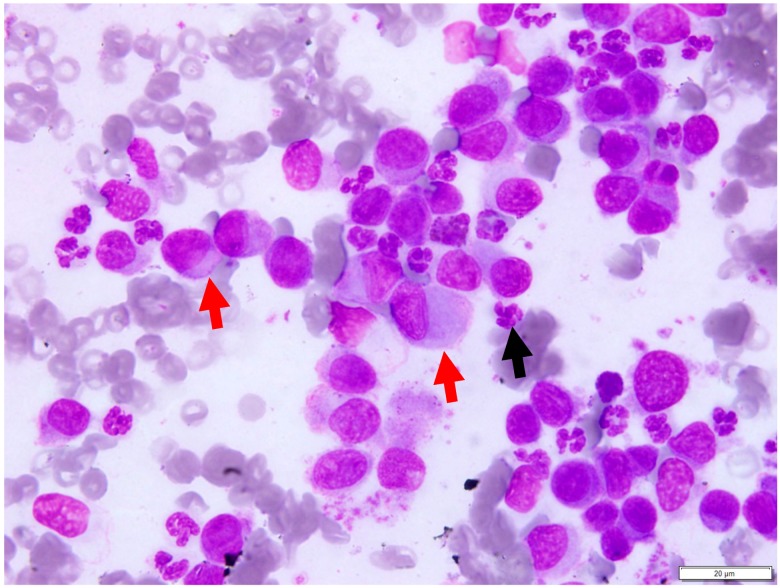
Fine needle aspirate of the oral mass. The slide consists predominantly of individually occurring, pleomorphic round cells with round to oval nuclei that are often eccentrically located within the cells (red arrows). Low numbers of neutrophils are also seen (black arrow); Wright-Giemsa Stain. Scale bar 20 µm, ×100 objective.

The individual slides were of low to high cellularity, and consisted of nucleated cells present in a background containing high numbers of red blood cells (Figure 1). The nucleated cell population consisted predominantly of individually occurring, round to oval cells with distinct cell borders and scant to moderate amounts of light basophilic cytoplasm. The nuclei were round to oval, often eccentrically located, and had a smooth to reticular chromatin pattern. Mild to moderate anisocytosis and anisokaryosis were observed. Occasional binucleated cells and low numbers of neutrophils were noted. These findings were suggestive of a plasma cell tumour, and histopathology was recommended for confirmation.

### 3.4. Histopathologic Findings

**Figure 2:**
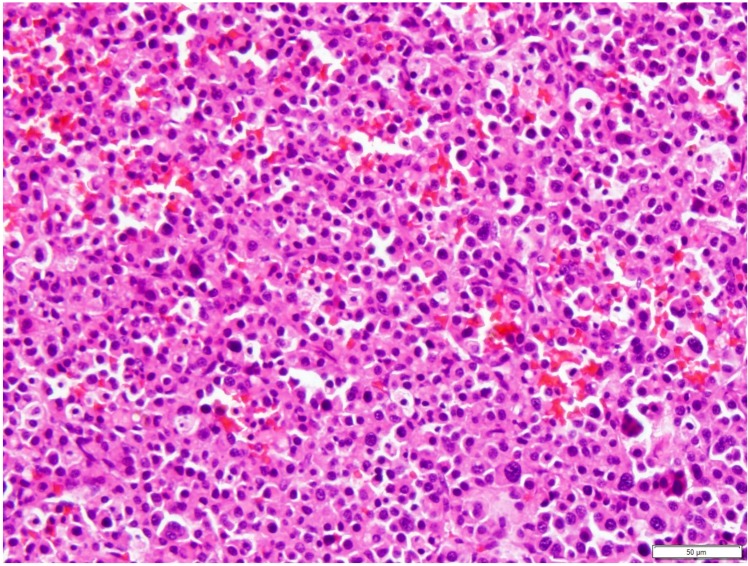
Histopathologic image of the mass in the oral cavity. The submucosa is replaced by large numbers of moderately densely packed round cells with moderate amounts of bright eosinophilic cytoplasm and an occasionally prominent Golgi apparatus. H&E stain. Scale bar 50 µm, ×40 objective.

The sections examined consisted of sheets and packets of moderately densely packed round cells on a thin fibrovascular stroma that extended to the deep border (Figure 2). The round cells had moderate amounts of bright eosinophilic cytoplasm and an occasionally prominent Golgi apparatus. The nuclei were eccentric and rounded, with clumped heterochromatin. Mitoses were 2 per 10 high-power fields (40×). These findings were compatible with a plasmacytoma. Immunohistochemistry was not performed.

## 4. Discussion

In a study of oral tumours in dogs over a 10-year period (1996–2006), the most common oral cancers were melanoma (30.7%), squamous cell carcinoma (18.2%), osteosarcoma (8.9%), and fibrosarcoma (8.9%). Epulides accounted for 18% of the oral masses, while oral EMP represented 5.2% of all oral tumours. Oral EMPs were found on the tongue, originating from the rostral aspect of the mandible, on the maxilla, hard palate, and on the buccal surface of the upper lip [6].

Approximately 50% of all human cases of EMP have hyperglobulinemia, and one in three will progress to multiple myeloma [7]. Serum electrophoresis was not performed in this case to determine if the hyperglobulinemia was due to a monoclonal gammopathy. However, it is more likely that the hyperglobulinemia was due to inflammation associated with the mass within the gingiva and was not a paraneoplastic syndrome, as hyperglobulinemia is not generally associated with EMPs in dogs [3,4,5].

In dogs, repeated studies have shown no obvious correlation between EMPs and the development of multiple myeloma. Unlike multiple myeloma, EMPs have a more favourable prognosis, tend to be locally invasive often causing osteolysis, which was seen in this case, but have a low rate of metastasis [4]. Multiple myelomas arise in an intramedullary location and usually result in osteolysis, however the principal locations in dogs include the vertebra, femur, pelvis, humerus, and ribs, with the jaw being an unlikely location [8]. A multiple myeloma originating in the marrow cavity of the bone and extending into the gingiva was therefore not considered a likely possibility in this case.

Dogs with a mucocutaneous EMP usually present with a single, visibly smooth, raised, red mass, which may be ulcerated or bleeding. There is evidence that suggests that a canine oral EMP may develop at sites of chronic inflammation such as gingivitis and periodontal disease [6,9]. This hypothesis is supported by the presence of mature T cells and dendritic cells within the oral EMP, suggesting both neoplastic and inflammatory components [9]. In this dog, the gross appearance of the mass, along with the presence of a dental disease (dental plaque on all the teeth), concurred with these findings. In humans, viruses, overdose irradiation, chronic irritation, and genetic disorders in the reticuloendothelial system have been suggested as the probable etiologic factors for the development of plasma cell neoplasms [9,10].

The diagnosis of plasmacytoma can be made by cytology or histopathology. Typical cytologic features are discrete round cells, eccentrically located nuclei, bi- and multinucleation, and variable amounts of basophilic cytoplasm with a perinuclear clear Golgi zone. The morphology of the cells in this case was similar to this typical morphology. Amorphous eosinophilic material, representative of amyloid, is seen in less than 10% of plasmacytomas [5,11].

Immunohistochemistry can be used for confirmation or for the identification of undifferentiated plasmacytomas, as other round cell tumours may have a similar morphological appearance [11]. The immunohistochemical diagnosis has historically been based on the demonstration of monoclonal lamda or kappa light chain reactivity, with 97–100% of all EMPs being positive for lamda light chain reactivity [12,13]. Canine plasmacytomas are also positive for IgG, IgA, or IgM, and for CD45, and variably express the leukocytic antigens CD18, CD45RA, and CD79a [14,15,16,17]. In dogs, CD79a is expressed in up to 80% of plasmacytomas [16,18]. Positive staining for Multiple Myeloma 1/Interferon Regulatory Factor 4 (MUM1/IRF-4) is also useful in the diagnosis of plasmacytomas. MUM1/IRF-4 is involved in lymphoid cell differentiation, particularly in the production of plasma cells. It is very specific for canine plasmacytomas and it allows the identification of canine plasmacytomas with a higher sensitivity and specificity compared with CD79a and CD20 [14,15].

Complete surgical excision is the primary treatment for oral and cutaneous EMPs and is often curative provided that there are no metastases [4,5]. Local recurrence has been reported, but the majority of these cases had neoplastic cells at the surgical margins of the excised specimens. In one study, dogs without complete surgical removal of an EMP and no adjuvant therapy had a median survival time of 138 days and tumour recurrence at a median time of 50 days [6]. As gingival EMP was found to have the highest recurrence rate, partial maxillectomy or mandibulectomy may be required to remove the entire tumour and prevent recurrence [5].

## 5. Conclusions

This paper describes a case of an oral plasmacytoma in a dog. This report also highlights the utility of cytology and histopathology in the diagnosis of neoplasms in situations where more advanced diagnostic tests such as immunohistochemistry are not available for further characterisation.

There are no case reports about this type of tumour in the Caribbean. This report represents the first from Trinidad. As more papers are published about the occurrence of round cell tumours such as plasmacytomas and lymphomas within the Caribbean, further studies can be done to see if there are geographical differences in the incidence between the tropical island countries, such as Trinidad, and other regions in the world, as well as the potential reasons for these differences.

## References

[1] Ettinger S., Feldman E. (2010). Textbook of Veterinary Internal Medicine.

[2] Borgatti A. (2010). Plasma Cell Tumors.

[3] Kupanoff P.A., Popovitch C.A., Goldschmidt M.H. (2006). Colorectal plasmacytomas: A retrospective study of nine dogs. J. Am. Anim. Hosp. Assoc..

[4] Rakich P.M., Latimer K.S., Weiss R., Steffens W.L. (1989). Mucocutaneous plasmacytomas in dogs: 75 cases (1980–1987). J. Am. Vet. Med. Assoc..

[5] Clark G.N., Berg J., Engler S.J., Bronson R.T. (1992). Extramedullary plasmacytomas in dogs: Results of surgical excision in 131 cases. J. Am. Anim. Hosp. Assoc..

[6] Wright Z.M., Rogers K.S., Mansell J. (2008). Survival data for canine oral extramedullary plasmacytomas: A retrospective analysis (1996–2006). J. Am. Anim. Hosp. Assoc..

[7] Nofsinger Y.C., Mirza N., Rowan P.T., Lanza D., Weinstein G. (1997). Head and neck manifestations of plasma cell neoplasms. Laryngoscope.

[8] Thompson K.G., Dittmer K.E., Meuten D.J. (2016). Tumors of Bone. Tumors of Domestic Animals.

[9] Schrenzel M.D., Naydan D.K., Moore P.F. (1998). Leukocyte differentiation antigens in canine cutaneous and oral plasmacytomas. Vet. Dermatol..

[10] Raskin R.E., Meyer D. (2010). Canine and Feline Cytology: A Colour Atlas and Interpretation.

[11] Withrow S.J., Vail D. (2001). Withrow & MacEwen’s Small Animal Clinical Oncology.

[12] Platz S.J., Breuer W., Pfleghaar S., Minkus G., Hermanns W. (1999). Prognostic value of histopathological grading in canine extramedullary plasmacytomas. Vet. Pathol..

[13] Cangul I.T., Wijnen M., Van Garderen E., van den Ingh T.S. (2002). Clinico-pathological aspects of canine cutaneous and mucocutaneous plasmacytomas. J. Vet. Med. A Physiol. Pathol. Clin. Med..

[14] Fernandez N.J., West K.H., Jackson M.L., Kidney B.A. (2005). Immunohistochemical and pathology. histochemical stains for differentiating canine cutaneous round cell tumors. Veterinary.

[15] Ramos-Vara J.A., Miller M.A., Valli V.E. (2007). Immunohistochemical detection of multiple myeloma 1/interferon regulatory factor 4 (MUM1/IRF-4) in canine plasmacytoma: Comparison with CD79a and CD20. Vet. Pathol..

[16] Gross T.L., Ihrke P.J., Walder E.J., Affolter V.K. (2005). Skin Diseases of the Dog and Cat: Clinical and Histopathologic Diagnosis.

[17] Ramos-Vara J.A., Miller M.A., Pace L.W., Linke R.P., Common R.S., Watson G.L. (1998). Intestinal multinodular A lambda-amyloid deposition associated with extramedullary plasmacytoma in three dogs: Clinicopathological and immunohistochemical studies. J. Comp. Pathol..

[18] Gholizadeh N., Mehdipour M., Rohani B., Esmaeili V. (2016). Extramedullary Plasmacytoma of the Oral Cavity in a Young Man: A Case Report. J. Dent. (Shiraz).

